# A Comprehensive Study of the Antibacterial Activity of Bioactive Juice and Extracts from Pomegranate (*Punica granatum* L.) Peels and Seeds

**DOI:** 10.3390/plants10081554

**Published:** 2021-07-28

**Authors:** Kaja Kupnik, Mateja Primožič, Katja Vasić, Željko Knez, Maja Leitgeb

**Affiliations:** 1 Laboratory for Separation Processes and Product Design, Faculty of Chemistry and Chemical Engineering, University of Maribor, Smetanova ulica 17, 2000 Maribor, Slovenia; kaja.kupnik@um.si (K.K.); mateja.primozic@um.si (M.P.); katja.vasic@um.si (K.V.); zeljko.knez@um.si (Ž.K.); 2 Faculty of Mechanical Engineering, University of Maribor, Smetanova ulica 17, 2000 Maribor, Slovenia; 3 Faculty of Medicine, University of Maribor, Taborska ulica 8, 2000 Maribor, Slovenia

**Keywords:** antibacterial, bioactive, enzyme activity, pomegranate, *Punica granatum*

## Abstract

Due to the growing awareness of *Punica granatum* fruit’s health-promoting properties, the pomegranate is increasingly used for food purposes. This results in the formation of biological waste products such as peels. A biowaste circular bioeconomy strategy holds great prospective for a sustainable economy. Therefore, a sustainable and environmentally friendly way of disposing of waste (e.g., use of biowaste to obtain high-value components (e.g., punicalagins, enzymes)) is crucial for the protection of the environment and human health. In the presented study, the content of total phenols and proanthocyanidins in ten samples of *Punica granatum* fruit (juice, aqueous (H_2_O) and ethanolic (EtOH) extracts of peels and seeds) was determined. Peel extracts were found to be the richest in the content of secondary metabolites and showed extremely high antioxidant potential (approximately 90% inhibition: DPPH radical scavenging activity). To the best of our knowledge, this is the first comparative study to determine the enzymatic activity of α-amylase, lipase, peroxidase, protease, and transglutaminase in different *P. granatum* samples. Furthermore, the antibacterial efficacy of all *P. granatum* samples was qualitatively determined against three strains of Gram-negative (*Escherichia coli*, *Pseudomonas aeruginosa*, and *Pseudomonas fluorescens*) and three strains of Gram-positive (*Bacillus cereus*, *Staphylococcus aureus*, and *Streptococcus pyogenes*) bacteria, susceptible to gaining antibiotic resistance. Moreover, the most promising peel extracts were quantified for antibacterial efficacy against tested bacteria at five different concentrations. All samples slowed down and inhibited the growth of all tested bacteria. MIC_90_ values (2.7 or 0.3 mg/mL) were determined in 18 out of 24 experiments (four samples, six bacteria tested). There is no research in the reviewed literature that is current with such detailed and comprehensive determination of *P. granatum* peel extracts antibacterial activity. The results of the research showed great potential for the use of *P. granatum* in the field of antibacterial activity in biomedicine applications and in the cosmetic, food, and pharmaceutical industries.

## 1. Introduction

Over the past few years, antimicrobial resistance (AMR) increased drastically and poses a major medical problem and challenge around the world [1]. According to the World Health Organization, AMR is one of the top ten global public health threats confronting humanity [2]. The increasing toxicity of synthetic drugs and, at the same time, the reduction in their effectiveness are just two of the additional reasons for concern. Hence, research in recent years has focused on finding new plant-based antimicrobials. The predisposition is excellent, as about 25–50% of current pharmaceuticals are already of plant origin [3].

Plants and their extracts have been exploited for many years in various branches of traditional medicine [4] and due to their abundance, can provide a wide range of secondary metabolites with structural diversity. Important building blocks of plants are enzymes, which play a key role in the production of biomolecules, and above all, show great potential as an effective source for the production of plant-based enzymes for use in various branches of industry and medicine [5].

The promising properties of plant secondary metabolites may be alternative sources in the fight against microbial resistance [6]. Phytoconstituents like alkaloids, flavonoids, polyphenols, tannins, and others could serve as resistance modifiers and potentials for antimicrobials. It is necessary to emphasize the ability of plant extracts to bind to protein domains, which can lead to inhibition or modification of protein-protein interactions. Therefore, plant materials and their extracts could be used as modulators of cellular processes, as they could kill the microbial cells and moreover reduce the ability of microorganisms to develop resistance to botanicals [7].

Due to the food process industry, there is a lot of fruit waste on an annual basis because raw fruits are processed into products that have increased added value. *Punica granatum*, better known as pomegranate, has become an interesting functional food in the last two decades [8]. About 50% of the weight of *P. granatum* fruit consists of inedible parts, i.e., the peel, while the remaining half consists of about 20% of the seeds and 80% of the juice [9]. Studies show that *P. granatum* fruit is rich in anthocyanidins, flavonoids, and phenolic compounds, but the phytochemical composition varies in different parts of the fruit and also depends on geographical and environmental factors that may affect the bioactivity level of secondary metabolites [8,10]. In addition to various micro- and macronutrients, and bioactive compounds present, sources indicate that *P. granatum* fruit possesses exceptional antioxidant properties, as well as provides anti-atherosclerotic, anti-hypertensive, anti-inflammatory, and anti-mutagenic properties, and has a positive effect on wound healing [11,12]. 

However, *P. granatum* fruit and its parts are most commonly used because of its antimicrobial properties [13]. Antimicrobial activity of *P. granatum* peels, seeds and juice has been demonstrated against various food and waterborne bacteria, and human pathogens, including *Escherichia coli, Bacillus subtilis, Candida albicans, Clostridium* spp., *Helicobacter pylori, Klebsiella pneumoniae, Listeria monocytogenes, Pseudomonas aeruginosa, Salmonella typhi, Shigella* spp., *Staphylococcus aureus, Vibrio cholerae, Yersinia enterocolitica*, and others [14,15,16,17,18,19,20,21].

**Figure 1:**
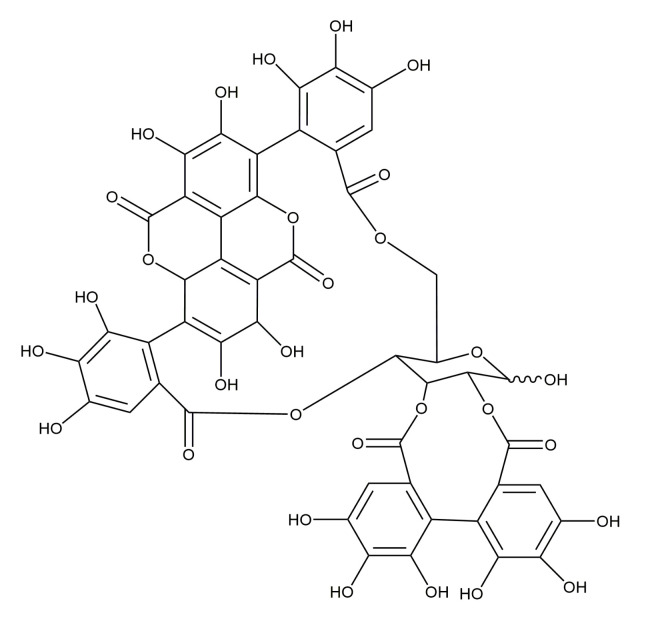
Chemical structure of punicalagins [21].

Different polyphenolics were successfully isolated and determined in pomegranate, namely punicalagins (Figure 1), punicalins, and other ellagitannins [22,23]. These constituents are acknowledged for their biological activity (antioxidant, anti-inflammatory, and antimicrobial) [24], while punicalagins have been shown to be the source of the antimicrobial activity of *P. granatum* peels [25].

Hence, peels treated as a food waste could be used as a valuable source for the isolation of punicalagins or enzymes, which could be further used in biomedicine, pharmaceutical, food, cosmetic and other industries.

The greatest contribution of this research is a comprehensive study of the enzymatic and antibacterial activity of the *P. granatum* samples. Singh et al. [26] recently provided an excellent review of the antimicrobial potential of *P. granatum* peels; however, our study is the first one to quantitatively determine the growth inhibition rate of Gram-negative and Gram-positive bacteria at five concentrations of four different extracts of *P. granatum* peels.

## 2. Results

### 2.1. Enzymatic Activity of P. granatum Fruit

In biotechnology and in the field of commerce, enzymes are used primarily as industrial catalysts, therapeutic agents, analytical reagents, and manipulative tools [27]. For this reason, the activity of various selected enzymes in *P. granatum* samples was determined for possible different applications. The results are shown in Table 1.

In terms of total protein content in *P. granatum* samples, seeds and peels had comparable concentrations (ranging from 0.0206–0.1264 mg/mL), while the highest total protein concentration was present in fresh *P. granatum* juice.

Furthermore, the enzymatic activity of α-amylase was also detected in the samples. Comparing the samples, α-amylase activity (0.0002–0.0119 U/mL) was determined in all *P. granatum* seed extracts, while the highest activity was detected in the EtOH extract of lyophilized peels (0.0676 U/mL). Its presence and isolations could be used for purposes in the brewing and starch industry, where α-amylases have been used for centuries [28].

Lipase activity (0.0492–0.4682 U/mL) was determined in the peel extracts, while it was not detected in the seed extracts. Fresh juice also showed 0.0328 U/mL lipase activity.

EtOH lyophilized peel extract, EtOH seed extract, and fresh juice also showed peroxidase activity (0.0020–0.1131 U/mL), whereas protease activity was detected only in the case of seed extracts (0.0121–0.0326 U/mL).

Interestingly, EtOH peel extract, H_2_O seed extracts and juices also showed transglutaminase activity (0.0166–0.3371 U/mL), the highest being detected in the case of *P. granatum* fresh juice.

The results of this study show great potential of different pomegranate fruit parts as plants have been of interest for many years for isolating enzymes. The activity of the aforementioned enzymes has been demonstrated in *P. granatum* samples, so it would make sense to use this potential for further utilization in nutritional supplements. Chidambara Murthy et al. [29] already determined levels of various enzymes (catalase, peroxidase, and superoxide dismutase) in extracts of *P. granatum* peels, but the results are not comparable due to the different detection methods and presentation of results in different units. To the best of our knowledge, no other references containing enzymatic activity in different samples of *P. granatum* have been detected. Therefore, the results of this study make a huge contribution to the identification of important enzymes from *P. granatum*.

### 2.2. Content of Other Secondary Metabolites in P. granatum Fruit

The extraction of phytochemicals or biologically active substances from plant sources is one of the important challenges for scientists. In our study, fresh and lyophilized juice of *P. granatum* fruit was produced. Moreover, aqueous and ethanolic extracts of various parts of *P. granatum* fruit were prepared. The content of important secondary metabolites were determined and antioxidative potential of the prepared samples were studied. The results of phytochemical studies are shown in Table 2.

As shown in Table 2 the total phenolics are most abundant in peels, followed by seeds, and juice. The highest value of TP was determined in EtOH peel extract, and a similar TP content was also present in the H_2_O lyophilized peel extract. While as expected, no TP content was detected in aqueous extracts of *P. granatum* seeds, TP were successfully extracted with ethanol. Lyophilized juice contained a higher content of total phenols than a fresh one, as with the lyophilization process the juice was concentrated, which is the reason for such a result. Nevertheless, lyophilized juice showed a lower TP content than seed and peel extracts, which is consistent with the literature, as it is known that the highest values of total phenols are found in the peels and seeds of *P. granatum* [30,31]. Li et al. [32] found that the TP content of the peel extract is approximately 10 times higher than that of the pulp, which coincides with results of present study.

The study showed the presence of proanthocyanidins in *P. granatum* samples. Ethanol generally extracted higher PAC contents than water. In the case of the ethanol as a solvent, higher PAC contents have fresh peel and seed extracts, while aqueous extraction yielded higher PAC values in lyophilized peels and lyophilized seeds. The highest PAC content was contained in EtOH seed extract, followed by EtOH peel extract, H_2_O lyophilized peel extract, EtOH extract of lyophilized seeds, and EtOH extract of lyophilized peels. Again, lyophilized juice contained higher PAC content than fresh juice.

The antioxidative potential of *P. granatum* samples has been investigated with DPPH^•^ radical scavenging activity. Peel extracts showed the highest antioxidant activity (three out of four peel extracts exhibited approximately 90% activity) among all samples. H_2_O peel extract achieved lower activity (approximately 14%), which can be associated with a lower content of secondary metabolites, as no PAC content was detected. In general, ethanol extracts possess a higher antioxidant potential compared to aqueous extracts, while seed extracts have a much lower antioxidative potential than the peel extracts, which is comparable to published studies [18,31,33]. However, for the assessment of the precise antioxidant capacity of the samples, it would be necessary to perform and select specific combination of methods with different mechanisms of action (e.g., oxygen radical absorbance capacity, chemiluminescence, Trolox equivalent antioxidant capacity, and ferric reducing antioxidant power among others [34]).

*P. granatum* fruits in the presented study were purchased at a local food store, so it should be taken into account that the samples were not made from freshly harvested fruits, and the storage and transport conditions themselves could affect the content of the analyzed metabolites. All investigated contents varied according to extraction solvent as well as parts of the plants. Results show that the highest values of TP and PAC were obtained with ethanol extraction.

### 2.3. Antibacterial Activity of P. granatum Extracts

Medicinal plants, used as natural remedies, offer the possibility of an alternative solution in the fight against antibiotic resistance. For this reason, the presented article includes a comprehensive and comparative study of the antimicrobial efficacy of *P. granatum* fruit. Our study complements and builds on many already published studies on this topic, as it includes a detailed analysis of the antibacterial activity of *P. granatum* fruit.

Preliminarily, the antibacterial efficacy of 10 different *P. granatum* samples (EtOH peel extract, EtOH lyophilized peel extract, H_2_O peel extract, H_2_O lyophilized peel extract, EtOH seed extract, EtOH lyophilized seed extract, H_2_O seed extract, H_2_O lyophilized seed extract, fresh juice, and lyophilized juice) was checked by disc diffusion method (DDM). In addition, the four most effective samples were selected based on DDM for which antibacterial efficacy was quantified. The microbial growth inhibition rate (MGIR) on Gram-negative (*E. coli, P. aeruginosa,* and *P. fluorescens*) and Gram-positive (*B. cereus, S. aureus,* and *S. pyogenes*) bacteria was determined by the broth microdilution method (BMD) at five different concentrations of samples.

#### 2.3.1. Gram-negative Bacteria

Three different strains of Gram-negative bacteria, *E. coli*, *P. aeruginosa,* and *P. fluorescens*, which are very susceptible to gaining antibiotic resistance [35,36], were exposed to *P. granatum* samples. Table 3 shows the results of qualitative determination of the antibacterial activity of samples with DDM.

As can be seen from Table 3, all samples showed inhibition of bacterial growth. Starting with the juice, fresh and lyophilized juice inhibited the growth of *E. coli* and *P. fluorescens*. Comparing both, lyophilized juice has been shown to be more effective against Gram-negative bacteria, since it also inhibited the growth of *P. aeruginosa* at the concentration of 10^6^ CFU/mL besides the growth of *E. coli* and *P. fluorescens* and showed larger inhibition zones in majority.

Regarding seed extracts, the growth of *E. coli* was most effectively inhibited by the EtOH extract of lyophilized seeds, as the inhibition zone (13 mm) also appeared at a higher concentration (10^7^ CFU/mL), where the remaining extracts did not inhibit *E. coli* growth. Growth of *P. fluorescens* was best inhibited by H_2_O seed extract (22 mm at 10^7^ CFU/mL), followed by EtOH seed extract and H_2_O lyophilized seed extract. *P. aeruginosa* was not susceptible to the presence of seed extracts.

The most effective inhibitors of the growth of Gram-negative bacteria are definitely peel extracts, as all samples inhibited the growth of all three bacteria at both initial bacterial concentrations. The most susceptible to the presence of peel extracts according to DDM is *P. fluorescens*, followed by *E. coli* and *P. aeruginosa*. The diameter of the inhibition zone decreased with higher concentration of bacterial culture. For comparison, Khan et al. [37] studied the antibacterial activity of *P. granatum* H_2_O and EtOH peel extracts against *E. coli* and *P. aeruginosa,* resulting in 22–25.5 mm inhibition zones at unknown bacterial concentration. Bigger inhibition zones are probably due to higher applied concentration of samples against bacteria or lower bacterial concentration.

The results are in accordance with the reviewed literature [15,38], which states the highest antimicrobial activity of *P. granatum* peels compared to seeds and juice.

Given the excellent qualitatively determined antibacterial activity of *P. granatum* peel extracts against Gram-negative bacteria, BMD was used for more accurate quantitative antibacterial efficacy at different concentrations of applied peel extracts.

**Figure 2:**
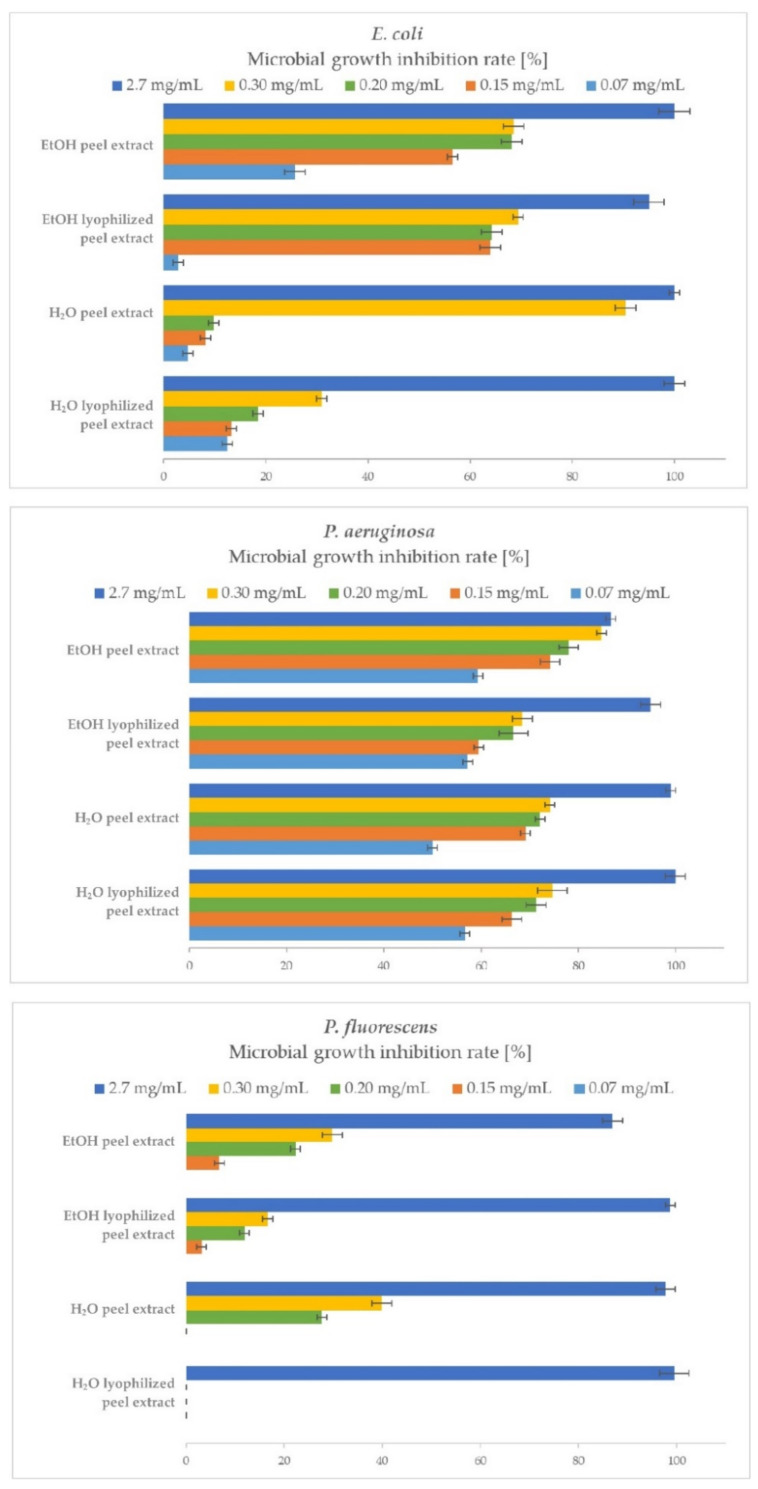
Microbial growth inhibition rates (MGIRs) for *P. granatum* extracts using 2.7, 0.3, 0.2, 0.15 and 0.07 mg of sample/mL of *E. coli*, *P. aeruginosa*, and *P. fluorescens* suspension. Initial concentrations of microorganisms were 1–5 × 10^6^ CFU/mL.

A comparison of the antibacterial efficacy and quantitative determination of the bacterial growth inhibition of *P. granatum* peel extracts on the growth of Gram-negative bacteria is shown in Figure 2.

In the present study, peel extracts proved to be good inhibitors of *E. coli* growth. At the highest added concentration (2.7 mg/mL) of both H_2_O peel extract and EtOH peel extract, *E. coli* growth was completely inhibited, resulting in 100% MGIR. EtOH lyophilized extract exhibited 95% MGIR. *E. coli* is more susceptible to the presence of EtOH extracts, as with the addition of lower concentrations, e.g., 0.15 mg/mL inhibited its growth by 57% and 64% MGIR compared to H_2_O extracts (8% and 13%).

Regarding the inhibition of *P. aeruginosa* growth, at the highest added concentration (2.7 mg/mL), the growth of *P. aeruginosa* was completely inhibited by both H_2_O peel extracts (100 and 99% MGIR), while the EtOH peel extract inhibited growth by 87% and the EtOH lyophilized peel extract with 95% MGIR. Moreover, it is important to point out that the samples very effectively inhibited the growth of *P. aeruginosa* even at the lowest added concentration of 0.07 mg/mL. In this case, the MGIRs ranged between 50–59%.

*P. fluorescens* was extremely susceptible to the addition of peel extracts at a concentration of 2.7 mg/mL, as EtOH lyophilized the peel extract and H_2_O extracts completely inhibited its growth (99, 98 and 100% MGIR). The EtOH peel extract inhibited *P. fluorescens* growth with 87% MGIR. Interestingly, lower concentrations of added samples did not show a good inhibitory effect, such as in the inhibition of *P. aeruginosa* growth. Additional studies should be performed to investigate the inhibitory effect of extract concentrations between 2.7–0.3 mg/mL.

The MIC_90_ values could also be determined from the obtained results. In the case of *E. coli*, the MIC_90_ value for H_2_O peel extract is 0.3 mg/mL and for other extracts, 2.7 mg/mL. MIC_90_ for both H_2_O extracts and EtOH lyophilized peel extract in the case of *Pseudomonas* spp. is 2.7 mg/mL, while for EtOH peel extract, additional studies above this concentration should be performed to determine the MIC_90_ value. For comparison, Negi et al. [39] determined the minimum inhibitory concentration (MIC) of H_2_O peel extract at 0.7 mg/mL for *E. coli* and 0.4 mg/mL for *P. aeruginosa*. Further, Nuamsetti et al. [16] found MIC for *E. coli* for H_2_O peel extract at 207 mg/mL, while for EtOH peel extract at 499 mg/mL. Sangeetha et al. [40] determined MIC for EtOH dried peel extract at 1 mg/mL for *E. coli* and *P. aeruginosa.* More recently, Elshafie et al. [19] found that 10 mg/mL of H_2_O extract of dried *P. granatum* peels inhibited the growth of *E. coli* by approximately 60%; however, the results are not completely comparable with our study, as their growth inhibition was determined by DDM. No other comparable data is available in the literature.

According to the literature reviewed, no studies cover quantitative determination of MGIR of *P. granatum* peel extracts on Gram-negative bacteria. Therefore, our study gives credence and added value to *P. granatum* fruit for its antibacterial activity. Overall, all tested samples of *P. granatum* peel extracts showed exceptional antibacterial efficacy against Gram-negative bacteria, especially in inhibiting the growth of *P. aeruginosa*.

Beside susceptibility to gaining antibiotic resistance, *P. aeruginosa* is also the most common Gram-negative bacterium in cosmetic products [41], so it is important to emphasize the potential of obtained *P. granatum* extracts that could be used in cosmetics, as in addition to excellent antibacterial properties they also contain TP and PAC and showed good antioxidant potential.

#### 2.3.2. Gram-Positive Bacteria

*B. cereus, S. aureus*, and *S. pyogenes* are representatives of Gram-positive bacteria known for their antibiotic resistance [42,43,44]. The antibacterial efficacy of *P. granatum* samples on the growth inhibition of the aforementioned bacteria was tested. The results of qualitative DDM are presented in Table 4.

In the case of Gram-positive bacteria, the DDM showed inhibitory properties of all *P. granatum* samples. As the bacterial concentration increased, the zone of inhibition decreased.

Lyophilized juice inhibited growth of all three bacteria, while fresh juice was an effective antibacterial agent against *B. cereus*.

All seed extracts successfully inhibited the growth of *B. cereus* at both initial concentrations, while growth of *S. aureus* and *S. pyogenes* (10^6^ CFU/mL) was only inhibited by EtOH lyophilized seed extract.

Again, all Gram-positive bacterial cultures were most susceptible to the presence of peel extracts. The largest zones of inhibition occurred against *B. cereus* (21–23 cm), the growth of which was not inhibited only by H_2_O peel extract. *S. aureus* was most susceptible to EtOH lyophilized peel extract, followed by H_2_O peel extract, H_2_O lyophilized peel extract, and EtOH peel extract. All four peel extracts also successfully inhibited the growth of *S. pyogenes* at a concentration of 10^6^ CFU/mL (11–13 cm).

Comparing results to already published articles, some aqueous peel extracts did not show antibacterial activity against *S. aureus* [14], while in general, the peel extracts better inhibited the growth of Gram-positive bacteria as seed extracts and *P. granatum* juice [15,38,45].

Furthermore, BMD was used for quantitative antibacterial efficacy of *P. granatum* peel extracts at different concentrations.

**Figure 3:**
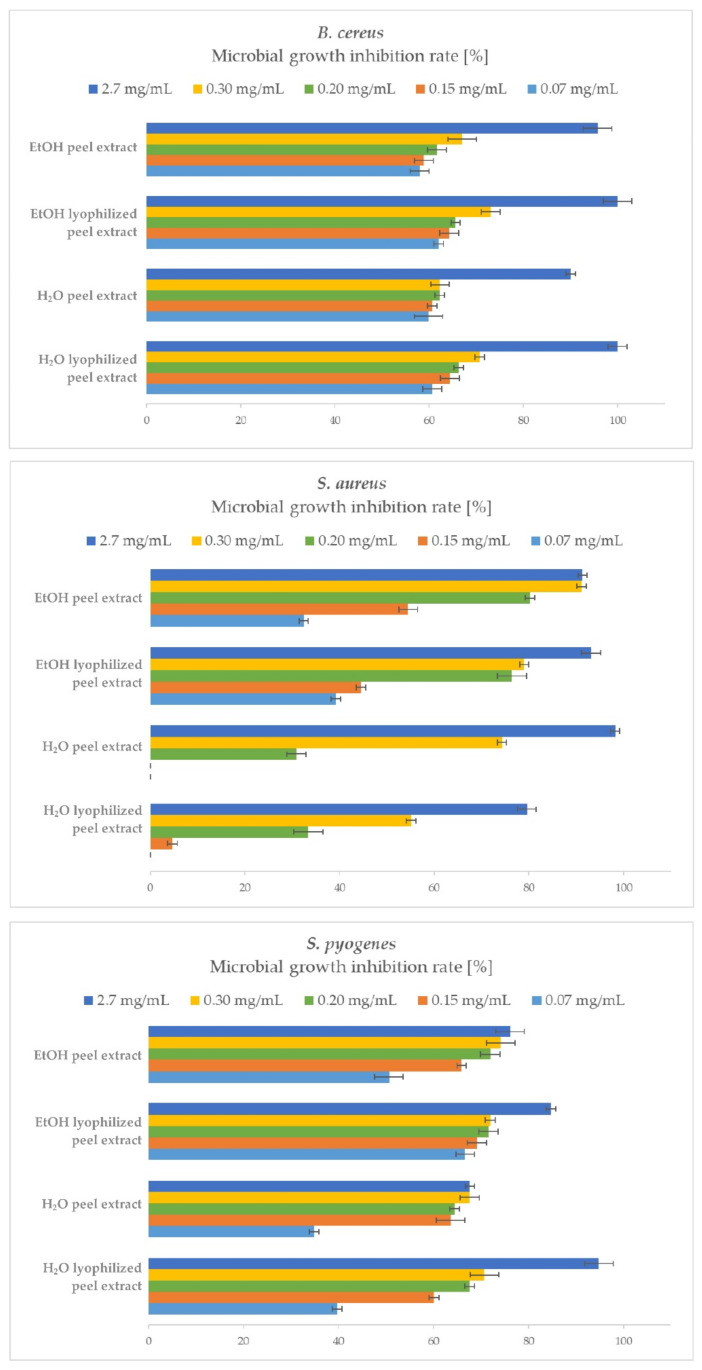
Microbial growth inhibition rates (MGIRs) for *P. granatum* extracts using 2.7, 0.3, 0.2, 0.15, and 0.07 mg of sample/mL of *B. cereus*, *S. aureus*, and *S. pyogenes* suspension. Initial concentrations of microorganisms were 1–5 × 10^6^ CFU/mL.

A comparison of the antibacterial efficacy and quantitative determination of the bacterial growth inhibition of *P. granatum* peel extracts on the growth of Gram-positive bacteria is shown in Figure 3.

Quantitative analysis of antibacterial activity of *P. granatum* peel extracts against Gram-positive bacteria was successfully performed. In the case of *B. cereus,* all samples exhibited strong antibacterial effects, since the H_2_O and EtOH lyophilized peel extracts completely inhibited its growth, while the H_2_O and EtOH peel extracts achieved 90 and 96% MGIR, respectively. It is important to note that even lower concentrations successfully inhibited the growth of *B. cereus*; for example, at a concentration of 0.07 mg/mL, the rates of inhibition of *B. cereus* growth ranged between 58–62%.

*S. aureus* was also very susceptible to higher concentrations of the added samples. Here, EtOH extracts proved to be more effective antibacterial agents, as H_2_O extracts at added lower concentrations (0.07 and 0.15 mg/mL) did not show any growth inhibition. At the highest sample concentration (2.7 mg/mL) added, *S. aureus* growth was best inhibited by H_2_O peel extract (98% MGIR), followed by EtOH lyophilized peel extract (93% MGIR) and EtOH peel extract (91% MGIR). The latter proved to be just as effective at nine times lower concentration (0.3 mg/mL), achieving the same growth inhibition rate (91% MGIR) as at the highest added concentration.

The growth of *S. pyogenes* was effectively inhibited by *P. granatum* peel extracts. The highest MGIR was achieved by the H_2_O lyophilized peel extract, resulting in 95% MGIR at 2.7 mg/mL, following the EtOH lyophilized peel extract (85% MGIR), EtOH peel extract (76% MGIR), and H_2_O peel extract (68% MGIR). In general, in terms of comparing samples, the lyophilized peel extracts were more effective in inhibiting the growth of *S. pyogenes* as peel extracts at the highest added concentration (2.7 mg/mL) of samples. On the other hand, comparing ethanol and water as an extraction solvent, EtOH extracts performed better at lower added concentrations (0.3, 0.2, 0.15, 0.07 mg/mL), inhibiting the growth of *S. pyogenes* more effectively with higher MGIRs than H_2_O extracts.

Moreover, MIC_90_ values have also been determined against Gram-positive bacteria. Regarding the growth inhibition of *B. cereus*, the MIC_90_ for all peel extracts was determined at 2.7 mg/mL. In the case of *S. aureus,* the MIC_90_ value for H_2_Opeel extract and EtOH lyophilized peel extract was 2.7 mg/mL, while for EtOH peel extract, a nine-times lower concentration (0.3 mg/mL) was needed to inhibit the growth of *S. aureus* by at least 90% MGIR. The MIC_90_ value has been determined also for H_2_O lyophilized peel extract against *S. pyogenes* at 2.7 mg/mL, while for another three samples, further studies should be performed to determine MIC_90_.

Some studies determined comparable MIC values for aqueous and ethanolic extracts of *P. granatum* peels. MIC of H_2_O peel extract was determined at 0.4 mg/mL for *B. cereus* [39] and from 0.45–104 mg/mL for *S. aureus* [16,39]. For the EtOH extract of the peel, the MIC was determined from 0.25–242 mg/mL for *S. aureus* [16,46]. The MIC for EtOH dried peel extract was for *S. pyogenes* determined at 0.5 mg/mL [40]. Other studies covering MIC values for *P. granatum* peel extracts have been performed primarily on methanol or other extracts and are not comparable to our results.

Again, no studies in the reviewed literature cover quantitative determination of microbial growth inhibition rate of *P. granatum* peel extract on Gram-positive bacteria. Hence, our study is the first to include such detailed information on the antibacterial efficacy of *P. granatum* peel extracts and give them additional credibility in terms of antibacterial activity.

### 2.4. The Most Promising P. granatum Samples for Different Applications

The results of the study are extremely promising, as with the highest added concentration (2. 7 mg/mL) in 18 cases (almost), complete growth inhibitions (at least 90% MGIRs) of bacteria were achieved. In addition, in the remaining six cases, the effective growth inhibition of bacteria was also detected (68–87 ± 3% MGIRs). Table 5 shows the determined MIC_90_ values for all tested *P. granatum* peel extracts.

Among Gram-negative bacteria, *E. coli* is most susceptible to H_2_0 peel extract with only 0.3 mg/mL, while *S. aureus* (Gram-positive bacteria) is most susceptible to EtOH peel extract with 0.3 mg/mL. The susceptibility of *P. aeruginosa*, a representative of Gram-negative bacteria, and *B. cereus*, a representative of Gram-positive bacteria, to *P. granatum* peel extracts should also be emphasized. All tested samples successfully slowed and inhibited the growth of the aforementioned bacteria, resulting in 50–62 ± 3% MGIRs even with the lowest applied concentrations (0.07 mg/mL).

To round out the results of our study, Table 6 shows the three most promising *P. granatum* samples for further applications in various fields of bioengineering.

Of all the samples, the sample with the most potential is the EtOH peel extract, which contains the highest concentrations of TP and PAC and exhibits the highest antioxidant potential. It also contains lipase and transglutaminase and has been shown to inhibit the growth of all Gram-negative and Gram-positive bacteria tested. EtOH lyophilized seed extract is overall the most promising in the case of seed extracts; when compared to EtOH peel extract, the content of TP and PAC and antioxidant potential is lower. The presence of α-amylase and protease was detected, and it inhibited the growth of *E. coli* and all of the tested Gram-positive bacteria. The lyophilized juice showed the best potential among juice samples, with the inhibition of all tested bacteria and the presence of α-amylase and transglutaminase.

## 3. Discussion

A comprehensive and comparative study of the antibacterial activity of biologically active samples from *P. granatum* peels, seeds, and juice was performed. In addition, different studies demonstrate the antimicrobial and antioxidant effect of various pomegranate extracts and report the isolation of various compounds responsible for *P. granatum* bioactivities [8,47]. Compounds like anthocyanin, caffeic acid, catechin, chlorogenic acid, cinnamic acid, coniferyl, coumaric acid, cyanidin, delphinidin, ellagic acid, flavonoids, gallic acid, genistein, kaempferol, linoleic acid, luteolin, pelletierine alkaloids, punicalin, punicalagin, quercetin, rutin, sinapyl, and tannin are responsible for significant antidiabetic, antihypertensive, antioxidant, antimicrobial, and other biological activities of pomegranate fruit [48].

Therefore, the content of total phenols and proanthocyanidins was determined in the samples. Among all tested samples, the extracts of *P. granatum* peels stand out, which contained the highest content of secondary metabolites. These extracts have also shown remarkable antioxidant potential. *P. granatum* grows in various parts of the world; its native land is Iran, while it is also found in India, Spain, the USA and the countries of the Middle and Far East [49,50]. Due to the cultivar type, climatic and growth locations/conditions, maturity, agricultural practices, temperature, storage, and transport conditions, plus other important factors, the content of bioactive constituents and their properties can be affected [50,51,52,53]. 

In recent years, a great deal of research has been published in the literature investigating the contents of polyphenols, proanthocyanidins, and antioxidant activity. It should be emphasized that various methods have been used for determination of biologically active compounds and their activities [54]. Due to all these factors, it is difficult to compare the results of studies directly with each other, especially because they are presented in different units (e.g., expressed per fresh weight, dry weight, extracts/sample, …) and determined by divergent procedures. Of particular importance is the selection, handling, and preparation of plant material for extraction, the choice of the right solvent, as well as the extraction process itself to prevent contamination of the extract and decomposition of important metabolites.

Additionally, the levels of various enzymes that are important in many branches of biotechnology have been determined in peels, seeds and juice from *P. granatum*. Plant lipases, peroxidases and proteases isolated from *P. granatum* peels could be utilized as an alternative to conventional chemical syntheses in industry, for example, in pharmaceuticals, textiles, food, detergents, cosmetics, biosensors and water treatment [55,56,57]. However, the enzymes present in *P. granatum* juice are important primarily for consumption and have a generally positive effect on human health, as some clinical applications are already known, especially for antioxidant enzymes [58]. As transglutaminases may be considered as wound-healing mediators [59], it would be interesting to test the synergistic effect of different bioactive components in such plant extracts, which also have antimicrobial properties for further applications in wound healing, drug delivery and topical use in the cosmetics, pharmaceutical industries, and biomedicine. Moreover, enzymology will continue to be considered as an area of active research, as the discovery of enzymes in new natural sources and their functional significance are demonstrated by a number of new studies and potential applications for these biocatalysts. Of particular interest is also the use for humans. Many nutritional supplements contain enzymes that are beneficial to general human health. In particular, amylases are added to help digest starch carbohydrates, proteases to help digest proteins into amino acid building blocks, and lipases to help break down fats into energy-rich fatty acids.

To substantiate the antibacterial efficacy of *P. granatum* samples, a qualitative diffusion method was used to confirm the growth inhibition of both Gram-negative and Gram-positive bacteria using pomegranate juice as well as peel and seed extracts. For a further, more comprehensive, and detailed study of antibacterial efficacy, the microdilution method was used to quantify the growth inhibition rates of microorganisms at different concentrations of the added inhibitory agent, *P. granatum* peel extracts.

Multi-drug resistant microorganisms are a major problem threatening human health around the world. Therefore, one of the most researched is the area of finding alternative solutions for the use of antibiotics [60]. According to the literature [39,61,62,63], Gram-positive bacteria are more susceptible to antimicrobial agents, as Gram-negative bacteria, due to their outer lipopolysaccharide membranes, are more resistant. Therefore, in our study, *P. granatum* extracts were tested precisely against Gram-negative and Gram-positive bacteria that are quickly adaptable and well-known to develop resistance. This is the first study with such extensive research to quantify the antibacterial efficacy of *P. granatum* peel extracts and makes a major contribution to science in the field of antibacterial activity. Due to the highest TP and PAC contents, the highest antioxidant potential and the exceptional inhibition of growth of all tested bacteria, the greatest potential among all samples is shown by EtOH peel extract.

In many parts of the world, pomegranates have been used in traditional medicine for centuries to treat many afflictions such as atherosclerosis, diabetes, various types of cancer, hypertension, ulcers, and oral diseases. However, it is currently gaining ground as a natural remedy and as a dietary supplement [64]. Our research further reinforced the fact that especially *P. granatum* peel extracts are effective agents with antibacterial activity and antioxidative potential. The extracts of *P. granatum* peels or isolated antibacterial compounds could be used further in the biomedicine, pharmaceutical, or cosmetic industry for various applications, for example, its incorporation in nanocellulose antimicrobial hybrids, which have been of great interest in recent years for further applications in the field of wound healing and targeted drug/antimicrobial delivery [65].

According to the results of the presented study, we see an exceptional potential, especially in *P. granatum* peels, as they are treated as waste food or as a by-product in the production of pomegranate juice. The extraction of bioactive components, especially those with antimicrobial activity, from *P. granatum* peels could thus be exploited to obtain pharmaceuticals and help to combat antibiotic resistance. On the other hand, such use of food waste could contribute to the circular economy and sustainable development. Sustainable biowaste management (e.g., use of peels of fruits for production of biocomponents with high-added value) will substantially contribute to the objective of halving the amount of residual municipal waste in EU by 2030, as proposed in the 2020 circular economy action plan [66].

## 4. Materials and Methods

### 4.1. Plant Material and Sample Preparation

Three pieces (total weight of 747.85 g) of *P. granatum* fruits (country of origin Spain) were obtained from the local food store. The fruit was peeled and arils were extracted. Further, the peels (358.79 g; 48% of total fruit weight) and arils (388.88 g; 52% of total fruit weight) were ground individually using homogenizer (Tehtnica^®^ Rotamix 701 MD, Železniki, Slovenia). The arils mixture was centrifuged (Eppendorf^®^ Centrifuge 5840R, Wesseling, Deutschland) for 5 min at 11,000 rpm and 20 °C. After centrifugation, the supernatant (*P. granatum* juice) (322.77 g; 43% of total weight) and the remaining ground seeds (66.11 g; 9% of total weight) were obtained. Part of the obtained juice, peels and seeds were lyophilized (Kambič^®^ Freeze Dryer LIO 2000 PNS, Semič, Slovenia).

### 4.2. Preparation of Extracts

Fresh and lyophilized peels and seeds were used to obtain *P. granatum* extracts.

#### 4.2.1. Preparation of Aqueous Extracts

The homogenizer-assisted extraction (HAE) [67] was used for the extraction of bioactive compounds from *P. granatum* fresh and lyophilized peels and seeds. For one batch, 5 g of lyophilized peels/seeds or 25 g of fresh peels/seeds were mixed with 150 mL of deionized water and homogenized for 1 h. Once the extraction was completed, the supernatant was separated from the insoluble solids by centrifugation for 5 min at 11,000 rpm and 20 °C. Supernatants (H_2_O extracts) were used for further analysis. Samples were stored at 4 °C until use.

#### 4.2.2. Preparation of Ethanolic Extracts

The Soxhlet ethanol extraction (SEE) [68] was used for the extraction of bioactive compounds from *P. granatum* fresh and lyophilized peels and seeds. For one batch, 5 g of lyophilized peels/seeds or 25 g of fresh peels/seeds were added to the porous cellulose thimble and placed in the extraction apparatus. Further, 150 mL of ethanol was added to a round bottom flask, attached to Soxhlet extractor and condenser. A heating mantle was used to reflux the mixture until the condensate was colorless. The extractions were conducted for approximately 4 h. The solvent was further evaporated with a rotavapor (Büchi^®^ Rotavapor R-144, Flawil, Switzerland). Ethanol extracts were used for further analysis. Samples were stored at 4 °C until use.

#### 4.2.3. Applied Concentrations of Samples

Since lyophilized juice of *P. granatum* was too dry for use in the experiments, it was diluted in deionized H_2_O to a concentration of 85 mg/mL. Ethanol extracts were diluted in 5% DMSO and applied with a concentration of 80 mg/mL. The obtained aqueous extracts, however, were of good consistency and were used undiluted in the experiments.

### 4.3. Chemical and Reagents

Chemicals including 100% acetic acid, 4-aminoantipyrine, Coomassie Blue G-250, ethanol, ferric chloride, hydrogen chloride, meat extract, meat peptone, n-butanol, 85% phosphoric acid, potassium dihydrogen phosphate, sodium chloride, sodium dihydrogen phosphate monohydrate, and sodium hydrogen phosphate were from Merck, Darmstadt, Germany. Acetonitrile, agar, amylose-remazol brilliant blue R, bovine serum albumin, casein, 3,5-dinitrosalicylic acid, 2,2-diphenyl-1-picrylhydrazyl, Folin-Ciocalteu’s Phenol-reagent, gallic acid, hydrochloric acid, hydrogen peroxide, 99% hydroxylamine hydrochloride, L-glutamic acid γ-monohydroxamate, L- Glutathione reduced, maltose, methanol, yeast extract, peptone from soybean, phenol, p-nitrophenyl butyrate, potassium sodium tartrate tetrahydrate, sodium acetate, sodium carbonate, starch, trichloroacetic acid, and Trizma Base were obtained from Sigma-Aldrich, Taufkirchen, Germany. CBZ-Glutaminylglycine was purchased from Zedira GmbH, Darmstadt, Germany. Potato dextrose agar and Mueller-Hinton broth were purchased from Biolife, Milano, Italy. Calcium chloride, D-(+)-glucose anhydrous, and iron (II) sulfate heptahydrate were obtained at Kemika, Zagreb, Croatia, while malt extract, potato dextrose broth, Triton X-100, tryptic soy broth, and tryptone were purchased from Fluka, Buchs, Switzerland.

### 4.4. Determination of Total Proteins and Enzyme Activities

#### 4.4.1. Total Protein Concentration

Protein concentration of *P. granatum* samples was determined by Bradford method using bovine serum albumin (BSA) as standard [69].

#### 4.4.2. Enzyme Activity Assays

Enzyme activities were measured by well-known spectrophotometric methods and were conducted in triplicate. α-Amylase activity was determined using amylose azure as substrate, as described by Leitgeb et al. [70]; one unit is expressed as the change in absorbance at 595nm/min/mL per solid of a-amylase at pH 7.0 at 35 °C. Lipase activity was measured using p-nitrophenyl butyrate (p-NPB) as substrate, described in the protocol by Sigma^®^ available at reference [71]; one unit will release 1 nmol of p-nitrophenol per minute at pH 7.2 and 37 °C using p-NPB. Peroxidase activity was measured according to the protocol by GoldBio^®^ described in the reference [72], using 4-aminoantipyrine (4-APP) as hydrogen donor; one unit will decompose one μM of H_2_O_2_ per minute at pH 7.0 at 25 °C. Protease activity was determined using casein as substrate. The protocol was previously described by Primožič et al. [73]: one unit is expressed as Tu^cas^ mL^−1^, which represents amount of casein hydrolyzed per mL of sample per minute. Transglutaminase activity was determined according to the protocol described by Gajšek et al. [74], using CBZ-Glutaminylglycine as substrate; one unit form 1 μmole of hydroxamate per minute at pH 6.0 and 37 °C.

### 4.5. Determination of Phenolics, Proanthocyanidins, and Antioxidant Activity

#### 4.5.1. Total Phenolic and Proanthocyanidin Content

The concentration of TP and PAC were determined by UV spectrophotometry protocols, described by Škerget et al. [75]. TP were determined based on a colorimetric reduction/oxidation reaction, using Ciocalteu’s reagent. The results are expressed as gram of gallic acid equivalents (GAE) per gram of extract/juice. PAC were determined based on color formation and acid hydrolysis. Hydrolysis of the samples was performed with an incubation time of 15 min. The results are expressed as mg of PAC per gram of extract/juice.

#### 4.5.2. DPPH^•^ Antioxidant Activity Assay

Antioxidant activity of *P. granatum* samples was determined based on 2,2-diphenyl-1-picryl-hydrazyl-hydrate (DPPH) free-radical method [76]. Briefly, 77 μL of prepared sample in methanol (1 mg/mL) was added to 3 mL of 6×10^−5^ M DPPH^•^ solution prepared in methanol. Gallic acid was used as a positive control. Mixtures were vortexed thoroughly and incubated in the dark for 15 min. Absorbance was measured at 515 nm against blank samples. The results are expressed as a percentage of inhibition [77].

### 4.6. Antibacterial Analysis

#### 4.6.1. Bacterial Cultures

The antibacterial activity of *P. granatum* samples was evaluated using the following strains of bacteria, Gram-negative bacteria: *Escherichia coli* (DSM 498), *Pseudomonas aeruginosa* (DSM 1128), *Pseudomonas fluorescens* (DSM 289), and Gram-positive bacteria: *Bacillus cereus* (DSM 345), *Staphylococcus aureus* (DSM 346), *Streptococcus pyogenes* (DSM 11728). These bacterial cultures were obtained from DSMZ-German Collection of Microorganisms and Cell Cultures GmbH, Berlin, Germany.

#### 4.6.2. Qualitative Analysis by Disc Diffusion Method (DDM)

Kirby-Bauer disc diffusion method, described by Kupnik et al. [78] was used for qualitative determination of antibacterial activity of *P. granatum* samples. 50 µL of potential inhibitor was applied on the 9 mm cellulosic disc. For negative control, the same amount of deionized H_2_0 and 5% DMSO were used, while as positive controls, amoxicillin and vancomycin (30 µg per disc) were used [79]. The experiments were performed in triplicates at two initial concentrations (10^6^ and 10^7^ CFU/mL) of bacterial cultures. The results are expressed in mm of the zone of inhibition that occurs around the sample on the inoculated nutrient medium.

#### 4.6.3. Quantitative Analysis by Broth Microdilution Method (BMD)

A broth microdilution method as described by Kupnik et al. [78] was used for the quantitative determination of antibacterial activity of the *P. granatum* samples. The medium used for the microdilution method was Mueller-Hinton broth. Initial concentrations of bacterial cultures were in the range of 1–5 × 10^6^ CFU/mL. MGIRs at different concentrations (0.07, 0.15, 0.20, 0.30, and 2.7 mg of sample per mL of bacterial suspension) of the added samples were determined. MGIRs reflect the percentage rate of inhibition of growth of a particular bacteria upon addition of a sample as an inhibitor and are determined based on the measured optical density (OD) of the sample and the OD of the bacterial growth control. Moreover, MIC_90_ were determined as concentrations at which the *P. granatum* extracts inhibited the growth of the microorganism by at least 90% MGIR. DMSO at a concentration of 5% served as a control for ethanolic extracts and deionized H_2_O was used as a control for water extracts. Results are presented as mean ± SD of three replicates.

### 4.7. Statistical Analysis

The data were analyzed using MS Excel (Microsoft 365) and presented as mean ± standard deviation of three replicates. Additional statistical analysis was carried out using Student’s *t*-test. Results were considered significant when *p* < 0.05.

## 5. Conclusions

The aim of this study was to investigate the enzymatic activity and content of bioactive constituents in *P. granatum* samples and to study their antioxidative potential and antibacterial activity with the aim to reduce the impact of biowaste on the environment. The study included 10 different samples of *P. granatum* fruit: ethanolic and aqueous extracts of fresh and lyophilized peels, ethanolic and aqueous extracts of fresh and lyophilized seeds, and fresh and lyophilized pomegranate juice. To the best of our knowledge, this is the first study to include α-amylase, lipase, peroxidase, protease, and transglutaminase activity in the samples tested.

This study revealed that *P. granatum* is a potential source of antibacterial compounds that are also effective against resistant strains of both Gram-negative and Gram-positive bacteria. All samples slowed down and inhibited the growth of all tested bacteria. MIC_90_ values (2.7 or 0.3 mg/mL) were determined in 18 out of 24 experiments. This is the first comprehensive report on the antibacterial activity of *P. granatum*, which includes quantification and determination of the growth inhibition rate of bacterial cultures at five different concentrations of 4 added samples.

The physicochemical and microbiological characteristics of peel and seed waste of *P. granatum* determine their various potential application possibility. Overall, it is necessary to highlight the EtOH peel extract, which is the most prominent in all respects. The presence of lipase and transglutaminase could be exploited for various applications in enzyme biotechnology, while the presence of total phenols and proanthocyanidins provides exceptional antioxidant potential and antibacterial activity of the extract. Peels could be exploited as a by-product of the food industry and as food waste to produce bioactive pharmaceutical ingredients for further use in the biomedicine, cosmetics and pharmaceutical industries and thus contribute to the circular economy and sustainable development.

Based on the current findings, it is recommended that the bioactivity of the individual identified bioactive compounds in *P. granatum* should be further investigated. Generally, the potential use of *P. granatum* peels and seeds for the recovery of bioactive-reach extracts is of pivotal importance within circular economy premises of production and utilization of natural resources. Additional studies of pomegranate peel and seed waste valorization should be performed since detailed explorations of the mechanisms and long-term risks of their valorization are still needed.

## Figures and Tables

**Table 1:** Activity of enzymes found in *P. granatum* extracts.

Sample	Total Proteins	α-Amilase	Lipase	Peroxidase	Protease	Transglutaminase
	(mg/mL)	(U/mL)				
EtOH peel extract	0.0206 ± 0.0008	n.d. ^1^	0.4682 ± 0.0069	n.d.	n.d.	0.1080 ± 0.0543
EtOH lyophilized peel extract	0.1264 ± 0.0031	0.0676 ± 0.0025	0.0492 ± 0.0015	0.0020 ± 0.0007	n.d.	n.d.
H_2_O peel extract	0.1005 ± 0.0084	n.d.	0.1570 ± 0.0244	n.d.	n.d.	n.d.
H_2_O lyophilized peel extract	0.1221 ± 0.0233	n.d.	0.0670 ± 0.0073	n.d.	n.d.	n.d.
EtOH seed extract	0.0230 ± 0.0003	0.0119 ± 0.0057	n.d.	0.1122 ± 0.0317	n.d.	n.d.
EtOH lyophilized seed extract	0.1177 ± 0.0458	0.0002 ± 0.0001	n.d.	n.d.	0.0326 ± 0.0101	n.d.
H_2_O seed extract	0.1000 ± 0.0195	0.0025 ± 0.0009	n.d.	n.d.	0.0136 ± 0.0084	0.0166 ± 0.0075
H_2_O lyophilized seed extract	0.1014 ± 0.0043	0.0014 ± 0.0001	n.d.	n.d.	0.0121 ± 0.0053	0.0209 ± 0.0049
Fresh juice	0.4409 ± 0.0317	n.d.	0.0328 ± 0.0076	0.1131 ± 0.0098	n.d.	0.3371 ± 0.0181
Lyophilized juice	0.1229 ± 0.0238	0.0009 ± 0.0004	n.d.	n.d.	n.d.	0.0048 ± 0.0007

^1^ n.d. — not detected. All values represent mean of three replicates ± standard error.

**Table 2:** Content of phenolics, proanthocyanidins in *P. granatum* samples and their antioxidative potential.

Sample	Total Phenols ^1,2^ (mg/g)	Proanthocyanidins ^3^ (mg/g)	Antioxidant Activity ^4^ (% Inhibition)
EtOH peel extract	24.0599 ± 2.5381	3.0549 ± 0.5145	90.4518 ± 3.7013
EtOH lyophilized peel extract	16.7613 ± 3.1294	1.3687 ± 0.1807	90.0512 ± 2.4588
H_2_O peel extract	13.5547 ± 1.0058	n.d. ^5^	14.2666 ± 1.0647
H_2_O lyophilized peel extract	23.3928 ± 1.9437	1.7882 ± 0.1234	90.3405 ± 3.2261
EtOH seed extract	15.8798 ± 2.4136	4.6710 ± 0.7951	15.8256 ± 1.2304
EtOH lyophilized seed extract	20.1662 ± 2.2164	1.5924 ± 0.0243	18.2951 ± 2.5168
H_2_O seed extract	n.d.	0.0035 ± 0.0011	10.1714 ± 3.4629
H_2_O lyophilized seed extract	n.d.	0.0068 ± 0.0029	14.0128 ± 1.6531
Fresh juice	3.6387 ± 0.1469	0.0755 ± 0.0076	5.3416 ± 0.9846
Lyophilized juice	7.5588 ± 0.5627	0.8406 ± 0.0143	7.6564 ± 1.3154

^1^ Concentration based upon gallic acid as standard. ^2^ Data in parentheses expressed as mg gallic acid equivalents (GAE) per gram of extract/juice. ^3^ Concentration expressed as mg of proanthocyanidins (PAC) per gram of extract/juice. ^4^ % DPPH^•^ radical scavenging activity. ^5^ n.d.—not detected. Values represent mean of three replicates ± standard error.

**Table 3:** Antibacterial activity of *P. granatum* samples on Gram-negative bacteria.

Sample	Diameter of the Inhibition Zone (mm)					
	*E. coli*		*P. aeruginosa*		*P. fluorescens*	
	10^6^ CFU/mL	10^7^ CFU/mL	10^6^ CFU/mL	10^7^ CFU/mL	10^6^ CFU/mL	10^7^ CFU/mL
EtOH peel extract	18 ± 2	16 ± 0	15 ± 1	14 ± 2	21 ± 1	20 ± 1
EtOH lyophilized peel extract	17 ± 1	16 ± 1	15 ± 1	13 ± 1	23 ± 2	20 ± 1
H_2_O peel extract	17 ± 1	13 ± 2	16 ± 2	14 ± 0	20 ± 0	19 ± 1
H_2_O lyophilized peel extract	18 ± 1	17 ± 1	16 ± 1	12 ± 0	21 ± 2	20 ± 1
EtOH seed extract	11 ± 0	/	/	/	13 ± 1	/
EtOH lyophilized seed extract	17 ± 1	13 ± 0	/	/	/	/
H_2_O seed extract	12 ± 2	/	/	/	24 ± 2	22 ± 1
H_2_O lyophilized seed extract	/	/	/	/	12 ± 1	/
Fresh juice	10 ± 1	/	/	/	12 ± 1	11 ± 0
Lyophilized juice	14 ± 1	/	16 ± 0	/	15 ± 1	11 ± 2

Data expressed as mean ± standard deviation of three replicates.

**Table 4:** Antibacterial activity of *P. granatum* samples on Gram-positive bacteria.

Sample	Diameter of the Inhibition Zone (mm)					
	*B. cereus*		*S. aureus*		*S. pyogenes*	
	10^6^ CFU/mL	10^7^ CFU/mL	10^6^ CFU/mL	10^7^ CFU/mL	10^6^ CFU/mL	10^7^ CFU/mL
EtOH peel extract	23 ± 2	20 ± 2	13 ± 2	/	13 ± 1	/
EtOH lyophilized peel extract	23 ± 2	21 ± 1	19 ± 1	14 ± 1	11 ± 1	/
H_2_O peel extract	/	/	13 ± 1	11 ± 1	12 ± 0	/
H_2_O lyophilized peel extract	23 ± 1	21 ± 0	13 ± 2	8 ± 2	12 ± 1	/
EtOH seed extract	12 ± 0	11 ± 0	/	/	/	/
EtOH lyophilized seed extract	13 ± 2	12 ± 0	12 ± 2	/	11 ± 0	/
H_2_O seed extract	14 ± 1	10 ± 0	/	/	/	/
H_2_O lyophilized seed extract	15 ± 2	11 ± 1	/	/	/	/
Fresh juice	16 ± 1	14 ± 1	/	/	/	/
Lyophilized juice	12 ± 2	11 ± 0	12 ± 0	/	11 ± 0	/

Data expressed as mean ± standard deviation of three replicates.

**Table 5:** Minimum inhibitory concentrations (MIC_90_) for *P. granatum* extracts on various bacteria.

Sample	MIC_90_ (mg/mL)					
	*E. coli*	*P. aeruginosa*	*P. fluorescens*	*B. cereus*	*S. aureus*	*S. pyogenes*
EtOH peel extract	2.7	> 2.7	> 2.7	2.7	0.3	> 2.7
EtOH lyophilized peel extract	2.7	2.7	2.7	2.7	2.7	> 2.7
H_2_O peel extract	0.3	2.7	2.7	2.7	2.7	> 2.7
H_2_O lyophilized peel extract	2.7	2.7	2.7	2.7	> 2.7	2.7

**Table 6:** The most promising *P. granatum* samples for further applications.

		EtOH Peel Extract	EtOH Lyophilized Seed Extract	Lyophilized Juice
Total phenols (mg/g) ^1^		24.0599 ± 2.5381	20.1662 ± 2.2164	7.5588 ± 0.5627
Proanthocyanidins (mg/g) ^2^		3.0549 ± 0.5145	1.5924 ± 0.0243	0.8406 ± 0.0143
Antioxidant activity (% inhibition) ^3^		90.4518 ± 3.7013	18.2951 ± 2.5168	7.6564 ± 1.3154
Enzymes present		lipase, transglutaminase	α-amylase, protease	α -amylase, transglutaminase
Proven inhibition of bacteria	Gram-negative	*E. coli, P. aeruginosa, P. fluorescens*	*E. coli*	*E. coli, P. aeruginosa, P. fluorescens*
	Gram-positive	*B. cereus, S. aureus, S. pyogenes*	*B. cereus, S. aureus, S. pyogenes*	*B. cereus, S. aureus, S. pyogenes*

^1^ Data in the parentheses expressed as mg GAE per gram of extract/juice. ^2^ Concentration expressed as mg of PAC per gram of extract/juice. ^3^ % DPPH^•^ radical scavenging activity.

## Data Availability

The data presented in this study are available in article.
